# Water Cooking Stability of Dried Noodles Enriched with Different Particle Size and Concentration Green Tea Powders

**DOI:** 10.3390/foods9030298

**Published:** 2020-03-05

**Authors:** Kun Yu, Hui-Ming Zhou, Ke-Xue Zhu, Xiao-Na Guo, Wei Peng

**Affiliations:** State Key Laboratory of Food Science and Technology, School of Food Science and Technology, Jiangnan University, 1800 Lihu Avenue, Wuxi 214122, Jiangsu, China; yukun472@yahoo.com (K.Y.); kxzhu@jiangnan.edu.cn (K.-X.Z.); xiaonaguo@jiangnan.edu.cn (X.-N.G.); pengwei@jiangnan.edu.cn (W.P.)

**Keywords:** dried green tea noodle, antioxidant activity, water cooking, microstructure

## Abstract

Incorporating green tea powder (GTP) into dried noodles enriched the functional characteristics of noodles. To achieve the maximum benefits from GTP, the water cooking stability of dried green tea noodles (DGTN) should be investigated. Indeed, antioxidant activities and phenolic compounds of DGTN after water cooking markedly decreased. The results showed that large GTP particles caused the increased cooking loss of DGTN, but the phenolic compound loss of DGTN prepared with them was low after cooking. Analysis of texture properties and microstructure showed that DGTN with a 2% concentration of large GTP particles formed some holes in the noodles’ network, and its breaking strength decreased. However, we observed that many GTP particles adhered to the surface of DGTN prepared with small GTP particles, and they were easier to lose after water cooking. Comprehensive analysis concluded that cooking loss, functional compounds retention and textural properties of DGTN were related to GTP particle size and concentration via the microstructure.

## 1. Introduction

Green tea is mainly produced in China, Japan and someeastern Asian countries, and usually consumed as tea beverages [1]. However, other hydrophobic active ingredients of green tea such as chlorophyll, insoluble protein and fiber, located inside the tea leaves, are hard to extract with water [2,3,4]. Green tea powder (GTP), which is the finely ground powder of leaves of green tea, has attained increasing attention and realized the whole utilization of green tea functional components. GTP can be used as a dietary supplement or flavoring materials in traditional foods such as noodles and bread [5,6,7,8].

Noodles are a kind of traditional cereal food in the Chinese diet, and many researchers have tried to combine them with GTP to meet the ever-growing nutritional demands of consumers. The textural and functional properties of green tea noodles have naturally become a hot topic of research. Previous studies on textural properties reported that the addition of GTP at a concentration of 2 g GTP/100 g wheat flour presented higher sensory scores according to color, and no significant difference was found for other sensory indicators among noodle samples with different levels of GTP [6]. Additionally, several studies on functional properties mostly focused on the changes of the phenolic compounds during processing, especially the stability of the tea catechins in green tea during baked food (such as bread and biscuit) processing and cooking [7,8]. To our knowledge, the stability of the phenolic compounds of green tea noodles during water cooking is still unknown.

However, extensive research has been therefore conducted to investigate the phenolic profile and antioxidant activity of pasta during the processing or boiling. The results consistently indicated that thermal treatment (boiling) caused a dramatic reduction of the total phenolic compounds and antioxidant activity [9,10,11,12]. In this regard, to achieve the maximum benefits from dried green tea noodles (DGTN), it is necessary to investigate the effects of GTP properties as well as the cooking stability of functional components on the retention rate of these beneficial components. In this study, the cooking loss and functional component leakage of DGTN affected by different particle sizes and concentrations of GTP were analyzed contrastively, and then the changes of textural and microstructure properties of DGTN subjected to particle sizes and concentrations of GTP were analyzed to elucidate the leakage.

## 2. Materials and Methods 

### 2.1. Materials

GTP 1, GTP 2 and GTP 3 were all kindly supplied by the Hangzhou Tea Research Institute of All-China Federation of Supply and Marketing Cooperatives. The measurement of GTP (shown in Table 1) and wheat flour protein content referred to the AACC Method 46-12.01, 2000a [13]. Wheat flour (9.95% protein, 13.69% moisture) was purchased at a local supermarket (Wuxi, Jiangsu, China) and stored at 4 °C for further experiments. 1, 1-diphenyl-2-picrylhydrazyl (DPPH) was purchased from Shanghai Yuanye Bio-Technology Co., Ltd. (Shanghai, China). Total antioxidant capacity assay kits including ABTS (2,2-azinobis-(3-ethylbenzothiazoline-6-sulfonic acid)) method and FRAP (ferric ion reducing antioxidant powder) method were purchased from Nanjing Jiancheng Bioengineering Institute (Nanjing, Jiangsu, China). Other reagents (Methanol, Acetone, Folin-Ciocalteu, Na_2_CO_3_) used in the analytical procedures were from Sinopharm Chemical Reagent Co., Ltd., China National Pharmaceutical Group Corporation (Wuxi, Jiangsu, China).

### 2.2. Particle Size Analysis and Color Measurement of GTP

Particle size distributions of GTP were determined by laser light scattering analyzer (S3500, Microtrac Inc., York, PA, USA). Adequate GTP samples were placed on the injection table, then the pneumatic sample injection device was started to push the sample into the test device. Particle size measurements were recorded as the volume mean diameter.

Color of GTP was analyzed by a Minolta CR-400 Chromameter (Model CR-400, Minolta Camera Co., Osaka, Japan) according to the standard of Commission Internationale de L’Eclairage L* a* b* (CIELAB) color system. L* (0 = black, 100 = white) represents the lightness. The reduction of a* values represents a color change from redness to greenness, and the increase of b* values represents a color change from blueness to yellowness. GTP was put on the sample plate and paved smoothly. Then, the color of the samples was measured by CR-400 Chromameter. Three parallel tests were carried at each sample.

### 2.3. Dried Green Tea Noodle Production

Each of the three GTPs was respectively mixed with wheat flour in weight ratios of 0.5%, 1%, 1.5% and 2% by using a small vacuum mixer (Model HWJZ-5, Nanjing Yangzi Grain and Oil Food Processing Machinery Co., Ltd., Nanjing, China). A 600 g amount of blended powder was mixed with 204 mL of deionized water to form a dough in a small vacuum mixer. After mixing for 7 min the dough was rested for 30 min at 25 °C and then was passed through an experimental noodle machine (Model JMTD-168/140, Dongfang Fude Technology Development Center, Beijing, China) for 13 times with the roller gap reduced gradually to get a 0.8 mm thickness noodle strands. The pre-defined drying program was referred to in the published article [14]. DGTN were dried through five stages of temperature time and humidity control by intelligent experimental equipment (Model SYT-030, China Packaging and Food Machinery Co., Ltd., Beijing, China), where the drying process was carried out at 36 (± 2) °C, 40 (±2) °C, 45 (± 2) °C, 40 (± 2) °C, and 30 (±2) °C, with a relative humidity of 80%, 70%, 60%, 60%, and 60% for 30, 40, 180, 30, and 30 min, respectively. Samples were dried to a final moisture content of 11%–12%. DGTN samples were cut into 20 cm length or freeze-dried and ground into 80-mesh powders according to the analysis requirements. Each DGTN sample was prepared three times.

### 2.4. Cooking Properties of DGTN

Cooking qualities of DGTN were determined according to the method of Inglett et al. [15] with minor modification. A 10 g sample of 20 cm in length DGTN was placed into 400 mL of boiling water until the optimum cooking time when the white core of noodles disappeared in boiling water. Meanwhile, the cooked DGTNs were drained with six filter paper, and then the weight was measured. Water absorption was expressed as the percentage increase in the weight of the cooked noodle compared to the weight of dried noodles. Noodle soup was collected in a 500 mL volumetric flask and diluted to the volume with distilled water, then 100 mL was taken into a 250 mL beaker pre-dried to a constant weight. The beaker was dried at 105 °C to a constant weight, and the residue was weighed. Cooking loss was calculated as a percentage of dry matter lost during cooking to dry DGTN weight. The measurements were carried out three times for each sample.

### 2.5. Measurement of Free Phenolic Content (FPC), Total Chlorophyll, Chlorophyll a and Chlorophyll b Contents

Determinations of FPC and chlorophyll contents were carried out as described in our earlier publications [14]. In brief, GTP (20 mg) and freeze-dried 80-mesh DGTN powder (0.2 g) were extracted in 4 mL of 70% methanol at 70 °C for 30 min and this process was repeated a couple of time. The extracts after centrifugation were collected and diluted to 10 mL with 70% methanol. A 1 mL volume of polyphenol extract was mixed with 4.5 mL of Folin-Ciocalteu reagent. After reacting 3 min, 5 mL of 7.5% Na_2_ CO_3_ solution was added and the reaction mixture was kept for 1 h in the dark. The absorbance was measured at 765 nm using a UV/VIS spectrophotometer (Model TU-1810, PERSEE, Beijing, China). The polyphenol content was quantified with gallic acid as a calibration standard.

The chlorophylls of GTP and freeze-dried 80-mesh DGTN powders were repeatedly extracted with 80% acetone until the color disappeared. The extracts were collected and made up to a certain volume. The absorbance of the chlorophyll extract was measured using a UV/VIS spectrophotometer at 663 and 645 nm, respectively. The calculation of chlorophyll content referred to our previous publications [14].

### 2.6. Antioxidant Activity

#### 2.6.1. Extraction

Freeze-dried 80-mesh DGTN powder (1 g) and GTP (20 mg) were respectively extracted according to the method of FPC in 2.5. Extract solutions of GTP and 2% DGTN samples were diluted four times with methanol before analysis. Extractions for testing were sealed in test tubes at −20 °C until the antioxidant analysis.

#### 2.6.2. 1-Diphenyl-2-Picrylhydrazyl (DPPH) Scavenging Activity

DPPH assay was measured according to the method of Savlak et al. [16] with some modification. The preparation of standard DPPH reaction solution: DPPH (6.4077 mg) was dissolved in methanol and completed to a 250 mL final volume to obtain a 0.065 mM DPPH solution. A 3.9 mL volume of DPPH solution was added to 100 μL methanol, reacted in the dark for 45 min. After the reaction, the absorbance value of this solution was about 1.2 ± 0.02 at 515 nm., which served as the control. Samples’ (DGTN powders and GTP samples) extracts (20, 40, 60, 80 and 100 μL) were completed with methanol to 100 μL final volume and mixed with 3.9 mL DPPH solution. Mixtures were placed in the dark for 45 min at room temperature. Then the absorbance value of sample ($A_{sample}$) was measured at 515 nm. Each sample was measured three times. The absorbance value of DPPH solution (3.9 mL) with 100 μL methanol served as control ($A_{control}$), and the absorbance value of methanol (3.9 mL) with 100 μL sample extract served as blank ($A_{blank}$). The formula for calculating the DPPH radical scavenging activity (RSA) was as follows:

(1) $$\mathrm{RSA}\left(\%\right)=A_{control}-\frac{A_{sample}-A_{blank}}{A_{control}}\times 100$$

The result was expressed as an IC_50_ value calculated from the graph of RSA percentages against extract concentrations.

#### 2.6.3. ABTS^•+^ Scavenging Activity

The scavenging ability against ABTS^•+^ was conducted referred to the operation instruction of total antioxidant capacity measurement kit (ABTS^•+^ assay, Item No.: A015-2-1, Nanjing Jiancheng Bioengineering Institute). In brief, peroxide solution was diluted 40 times with ultra-pure water into the application solution before it is used. ABTS^•+^ working solution was prepared by buffer solution, ABTS^•+^ solution and the application solution at a ratio of 76:5:4, and kept in the dark no more than 30 min. The peroxidase solution was diluted with buffer solution at a ratio of 1:9 before using. Trolox was used as standard substance. Trolox was dilute with pure water into different concentrations (0.1, 0.2, 0.4, 0.8, 1.0 mM). The reaction mixture consisted of 10 μL sample extract or different concentrations of Trolox, 20 μL diluted peroxidase solution, and 170 μL ABTS^•+^ working solution, then reacted for 6 min. The absorbance was read at 734 nm by using the Epoch 2 microplate spectrophotometer (Model EPOCH2T, BioTek Instruments Inc., Winooski, VT, USA). Trolox equivalencies of dry samples were calculated by the standard curve of Trolox (R^2^ = 0.99957). Results were expressed as mmol Trolox equivalents (TE)/kg dry DGTN sample or g dry GTP. Each sample was tested three replicates.

#### 2.6.4. Ferric Reducing Antioxidant Power (FRAP)

Ferric reducing antioxidant power (FRAP) was determined according to the operation instruction of total antioxidant capacity measurement kit (FRAP assay, Item No.:A015-3-1, Nanjing Jiancheng Bioengineering Institute). Briefly, FRAP working solution was prepared by buffer solution, matrix liquid and substrate solution at a ratio of 10:1:1, and kept in the dark at 37 °C no more than 2 h. Different concentrations of FeSO_4_·7H_2_O (0.15, 0.3, 0.6, 0.9, 1.2, 1.5 mM) were used for the calibration curve. The reaction mixture including 5 μL pure water (blank) or standard solution or sample extract and 180 μL FRAP working solution and then incubated for 5 min at 37 °C. The absorbance was read at 593 nm by using the Epoch 2 microplate spectrophotometer. FeSO_4_·7H_2_O equivalencies of dry samples were calculated by the standard curve of FeSO_4_·7H_2_O (R^2^ = 0.99712). Results were expressed as mmol FeSO_4_·7H_2_O equivalents/kg dry DGTN sample or g dry GTP. Each sample was tested three replicates.

### 2.7. Texture Analysis of DGTN

The breaking strength and elasticity of DGTN were measured using the Textural Analyzer (TPA; Stable Micro Systems, Godalming, UK) equipped with an A/SFR probe. Uncooked samples DGTN were cut into 10 cm, and one strand of uncooked DGTN was located between upper and lower supports in centrally located holes. The upper support is directly connected to the loaded and the lower support to the base of the texture analyzer. Measurements were carried out at room temperature. The average force and distance to break were measured. The test procedure was as follows: the pre-test speed, test speed, post-test speed, trigger force and distance were set to 2.00 mm/s, 2.00 mm/s, 10 mm/s, 5 g and 100 mm. The measurement values for each sample represented an average of ten times.

### 2.8. Scanning Electron Microscope (SEM) of GTP and DGTN

GTP was freeze-dried and sprinkled on double-sided adhesive tape mounted on specimen stubs. A piece of freeze-dried DGTN was hand-fractured and mounted on specimen stubs with the fractured side faced up. For observation of surface microstructure, freeze-dried DGTN was directly mounted on specimen stubs with the surface side face up. Samples mounted on specimen stubs were sprayed gold using a magnetron ion sputter metal coating device (MSP-2S, IXRF Systems, Inc., Japan). GTP and DGTN were observed at 5000, and 1000 magnification in 5.0 kV using a scanning electron microscope (SU8100, Hitachi High Technology Co., Ltd., Tokyo, Japan).

### 2.9. Statistical Analysis 

For each particle size of GTP, FPC, and chlorophyll content, the average value and standard deviation from three replicates of test results were figured out. Differences between means were determined by analysis of variance (ANOVA) with Tukey’s test, which were analyzed with SPSS 17.0 and a level of α < 0.05 was considered significant. All figures were drawn by OriginPro 2016 (OriginLab Corporation, Northampton, MA, USA).

## 3. Results and Discussion

### 3.1. Composition, Color, Particle size, and Microstructure of GTP

The composition, color and particle size of GTP are presented in Table 1. The particle size of GTP 1 and GTP 3 was significantly different compared with that of GTP 2. Cell diameter of a plant is usually in the range of 10–20 μm, so the particle size difference showed that the cell walls of these three kinds of GTP were damaged to varying degrees. The manufacture of GTP by superfine grinding technology could release the bioactive components by destroying cell walls to the greatest extent [17]. Bioactive components of GTP are mainly phenolic compounds. Obviously, the smaller the particle size of GTP, the higher the phenolic content.

**Figure 1:**
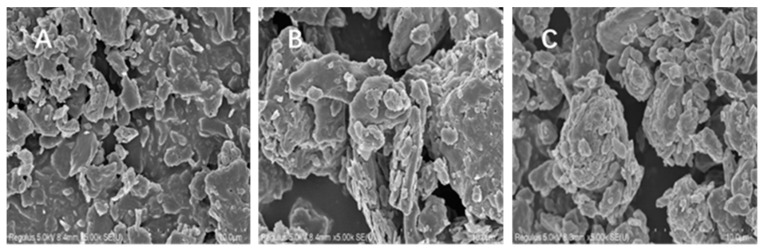
Scanning electron micrographs of GTP 1 (**A**), GTP 2 (**B**) and GTP 3 (**C**).

The SEM micrographs of the three GTPs are exhibited in Figure 1. Obviously, the observed microstructures of different particle sizes of GTP were significantly different. Generally, GTP particles occurred sticky and agglomerate due to electrostatic interaction and high specific surface area during superfine grinding processing. The results of GTP shapes we observed were consistent with previous research results [18] and displayed an irregular 3D structure. Moreover, compared with GTP 2 and GTP 3, on the particle morphology of GTP 1 appeared a state of aggregation and surface granulum adsorption. This phenomenon probably resulted from the larger specific surface of GTP 1, which was benefited by the cluster formation. By contrast, GTP 2 and GTP 3 aggregated to form large-scale particles and showed a distracted state.

### 3.2. Water Cooking Stability of DGTN

#### 3.2.1. Cooking Loss and Water Absorption

**Figure 2:**
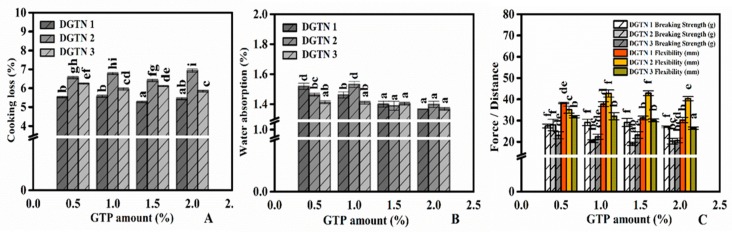
Effects of GTP with different particle sizes or concentrations on the cooking loss (**A**), water absorption (**B**) and breaking strength and elasticity (**C**) of dried green tea noodles (DGTN). Different lowercase letters show significant differences.

Cooking loss directly reflects the material loss of the dried noodles during water cooking. Figure 2A,B shows the effects of GTP particle size and concentration on the cooking properties of DGTN. Cooking loss of DGTN significantly increased with the increase of GTP particle size, while the concentration of GTP had no effect on cooking loss. Obviously, the particle size of GTP showed a greater negative effect on cooking loss than concentration. This might be due to that the large particle size of GTP could weaken the network of noodles resulting in the increase of starch granule loss. Additionally, the water absorption of DGTN notably reduced with the increasing particle size and concentration of GTP. It has been reported that wheat bran affected the transportation of water due to self-absorption during cooking [19], so we considered the self-absorption of GTP dietary fiber as a barrier in DGTN prevented water from diffusing in.

#### 3.2.2. Functional Component Leakages

The FPC and chlorophyll contents of DGTN with different particles size and concentrations of GTP are presented in Table 2. As expected, the FPC of GTP displayed positive effects on the FPC of DGTN. We observed that cooking caused a decrease in the FPC and chlorophyll contents of DGTN. A possible explanation for the decrease of FPC and chlorophyll content in DGTN might be the boiling water resulting in the degradation of chlorophylls and the leaching of phenolic compounds. There was an obvious difference in the retention rate of phenolic compounds and chlorophyll among DGTN with the different particle sizes and concentrations of GTP after cooking. The retention rate of phenolic compounds and chlorophyll decreased with the particle size of GTP decreasing. In addition, the retention rate of phenolic compounds in DGTN after water cooking increased with the concentration of GTP increasing, but chlorophyll had the opposite result. These results suggested that the loss mechanism of phenolic compounds and chlorophyll during cooking might be different. Therefore, we suspected that the loss of functional components during water cooking was not only related to the particle size of GTP but also to the texture properties of DGTN.

Noodle cooking is a process of thermal transfer and water absorption. The phenolic compound retention rate of DGTN 1 was significantly lower than that of DGTN 2 and DGTN 3. We speculated that the leaching of phenolic compounds during water cooking was probably related to the water absorption rate. Cooking water entered the internal structure of DGTN accompanying the dissolution of phenolic compounds. Indeed, the small particle size and low concentration of GTP caused the increase in the water absorption of DGTN. Besides, the particle size of GTP has an opposite effect on the retention rate of phenolic compounds and cooking loss. These results suggested that the loss of phenolic compounds did not play a role in the cooking loss, and the loss of phenolic compounds probably was related to the water migration during the cooking of DGTN. 

#### 3.2.3. Antioxidant Activity Changes

Firstly, comparisons in the antioxidant activity of the three GTPs determined by three different methods (DPPH, ABTS^•+^ and FRAP) are shown in Table 3. The correlation coefficient (R^2^) between IC_50_ and particle size of GTP was 0.6414, and their R^2^ were respectively −0.7806 and −0.5436 by the method of ABTS^•+^ scavenging activity and FRAP scavenging activity. As previously discussed, the bioactive components of GTP are better released when the cell wall of GTP is destroyed. Similar studies reported that the antioxidant activity owed more to phenolic compounds than others [20]. The antioxidant activity of GTP was also significantly correlated (*p <* 0.05) with total phenolic content, the correlation coefficients (R^2^) for IC_50_, ABTS^•+^ scavenging activity, and FRAP were, respectively, −0.8806, 0.9670 and 0.8166. Moreover, Savlak et al. [16] found that the effect of particle size on total phenolic content and antioxidant activity of unripe banana flour was statistically significant.

Changes in the antioxidant activity of DGTN before and after water cooking determined by three different methods (DPPH, ABTS^•+^ and FRAP) are shown in Table 3. The IC_50_ of DGTN was markedly decreased with the increase of GTP concentration. Whether DGTN with 0.5% GTP or 2% GTP concentration, the IC_50_ of DGTN before cooking was significantly affected by particle size distribution and, respectively, ranged from 188.5 to 224.3 mg/mL and 55.7 to 67.7 mg/mL. The DPPH scavenging activity of DGTN increased with the particle size of GTP decreasing. After water cooking the IC_50_ among different DGTN showed no significant difference. Therefore, we suspected that GTP with a smaller particle size caused a greater loss of antioxidant activity of DGTN after water cooking. It is known that green tea and its products are rich in natural antioxidants [21]. Therefore, the loss of antioxidant activity of DGTN was attributed to the thermal degradation of phenolic compounds by oxidation or polymerization [22]. Combined with the results of functional components leakage of DGTN after water cooking, we found that the phenolic retention rate of DGTN prepared with smaller particle size of GTP was indeed lower. With regard to the cooking process, the antioxidant capacity of DGTN determined by three different methods showed similar changes. Indeed, some researchers reported that ABTS ^• +^ scavenging activity and FRAP of products containing phenolic compounds increased due to the increase of phenolic content [23].

### 3.3. Texture Properties of DGTN

The results showed that the particle size of GTP did significantly influence the cooking loss of DGTN (Figure 2A). Other studies have suggested that cooking loss could be used to reflect the structural integrity of the noodles’ network [24]. Hence, we concluded that the particle size of GTP played an important role in the textural integrity of DGTN. Further, in order to understand the relationship between the particle size of GTP and the structure of DGTN, the breaking strength and flexibility that are close to the structure of DGTN are analyzed. The effects of GTP particle size and concentration on the breaking strength and flexibility of DGTN are shown in Figure 2C. Obviously, the breaking strength of DGTN decreased with the increase of GTP particle size and the breaking strength of DGTN prepared with GTP 2 and GTP 3 was decreased with the concentration of GTP increasing. Previous studies reported that the breakage susceptibility of dried spaghetti with the addition of bran decreased, and this result might be related to the dietary fiber in bran [19]. In this study, we speculated that the flexibility of DGTN was probably correlated with its breaking strength. When the breaking strength of DGTN was high, its flexibility would become worse. Previous studies found that cutting strength of textured vegetable protein with high GTP level increased owing to high density and hard extrudate, and appropriate GTP level improved microstructure of textured vegetable protein more orderly [25]. Therefore, textural property changes of DGTN with different particle sizes and concentrations of GTP would probably be attributed to the microstructure of DGTN. 

### 3.4. Microstructures of DGTN

**Figure 3:**
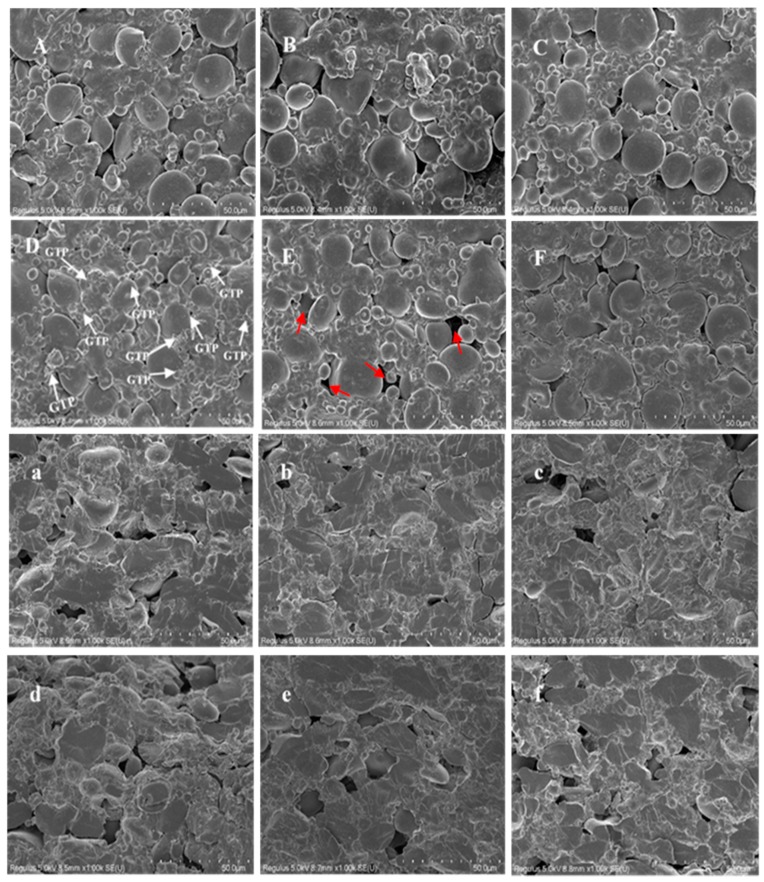
Scanning electron micrographs of DGTN with different particle sizes or concentrations GTP ((**A**–**F**) represent surface pictures of 0.5% DGTN 1, 0.5% DGTN 2, 0.5% DGTN 3, 2% DGTN 1, 2% DGTN 2, and 2% DGTN 3, respectively; (**a**–**f**) represent cross-sections of 0.5% DGTN 1, 0.5% DGTN 2, 0.5% DGTN 3, 2% DGTN 1, 2% DGTN 2, and 2% DGTN 3, respectively).

SEM micrographs of the surface (Figure 3A–F) and cross-section (Figure 3a–f) of uncooked DGTN with different particle sizes or concentrations of GTP can be cross-referenced to reveal the role GTP played in the preparation of DGTN. GTP has a higher content of protein and fiber than wheat flour, even though previous studies reported that protein and fiber had opposite effects on the noodles’ network [26]. Fiber broke the continuity of the protein–starch network but protein improved the protein–starch interactions. The microstructure among different DGTN showed no significant difference when the addition of GTP was 0.5%, however, when the concentration of GTP increased to 2%, the surface micrographs among different DGTN showed the discrepancy. As displayed in Figure 3D, we can observe many GTP particles (white arrow) attached to the surface of DGTN. However, there are hardly any GTP particles on the surface of 2% DGTN 2 and 2% DGTN 3. In addition, we can observe some big holes on the surface micrograph of 2% DGTN 2 (red arrow). This result proved that the larger particle size of GTP probably easily disrupted the noodles’ network. As previously discussed, we speculated that the larger particle size of GTP caused the big holes in the noodles’ network, and then increased the cooking loss of DGTN. The observed hole sizes in the surface micrograph of DGTN 2 were larger than 50 μm. However, the size of the A-Type granules size of wheat starch particles were 10–35 μm and the size of the B-Type granules size were <10 μm [27]. Therefore, the porous microstructure of DGTN 2 conduced to the leaching of starch granules during cooking. We observed a similar phenomenon on the cross-section of DGTN compared with the surface observation. Although DGTN 2 caused a larger cooking loss probably due to big holes in the network, its leakages of chlorophyll and FPC contents were lower than those of DGTN 1. These findings could be explained by the fact that cooking loss was more correlated with the loss of starch instead of the functional components. Therefore, we speculated that the leakage of functional components was more related to the particle sizes of GTP. Combined with the previous microstructure analysis of GTP and DGTN, we concluded that many small GTP 1 particles attached to the noodles’ surface were easier to leach from 2% DGTN 1 during cooking resulting in the decrease of retention rate. Therefore, from the perspective of functional characteristics, the smaller particle size of GTP was not conducive to the retention of functional components in DGTN.

## 4. Conclusions

The microstructure of small particle size GTP showed a state of aggregation, and some of these small particles adhered to the aggregation. Although large particle size of GTP increased cooking loss of DGTN, the phenolic retention rate of DGTN 1 due to that the small particle size of GTP 1 was resulted worse in a larger decrease of antioxidant activity after cooking. The increase of GTP particle size led to the decreased breaking strength. We observed the microstructures of DGTN and found many big holes in the starch–protein matrix. The large particle size of GTP caused starch granule exposure and resulted in the increase of cooking loss. Small GTP particles were easier to exist on the surface of DGTN and accelerated the leakage of GTP during cooking. Therefore, the suitable size of GTP particles is more beneficial to the texture properties and retention rates of functional components in DGTN. Further research can try to make GTP better wrapped in the starch–protein matrix, which may improve the retention rate of functional components.

## Figures and Tables

**Table 1:** Particle sizes, chemical components and color difference of green tea powders (GTPs).

-	GTP 1	GTP 2	GTP 3
Average volume particle diameter (μm)	6.60 ± 1.91 ^a^	15.53 ± 2.62 ^b^	9.78 ± 2.01 ^ab^
Protein (%)	24.43 ± 0.16 ^c^	22.62 ± 0.13 ^b^	20.32 ± 0.10 ^a^
Moisture (%)	4.19 ± 0.00 ^a^	5.88 ± 0.01 ^c^	5.08 ± 0.02 ^b^
Free phenolic (mg/g)	163.59 ± 0.21 ^c^	139.75 ± 0.11 ^a^	147.11 ± 0.09 ^b^
Chlorophyll *a* (*C_a_*/mg/g)	3.64 ± 0.03 ^c^	3.18 ± 0.02 ^b^	2.74 ± 0.06 ^a^
Chlorophyll *b* (*C_b_*/mg/g)	1.97 ± 0.01 ^b^	1.83 ± 0.01 ^a^	1.82 ± 0.04 ^a^
Total chlorophyll (TC/mg/g)	5.61 ± 0.05 ^c^	5.00 ± 0.08 ^b^	4.56 ± 0.10 ^a^
Color	-	-	-
L*	60.41 ± 0.00 ^b^	55.23 ± 0.20 ^a^	55.82 ± 0.41 ^a^
a*	-12.33 ± 0.01 ^a^	-9.01 ± 0.31 ^b^	-7.43 ± 0.03 ^c^
b*	27.07 ± 0.22 ^c^	25.08 ± 0.30 ^b^	24.12 ± 0.31 ^a^

Different lowercase letters show significant differences (α < 0.05) among the three GTPs.

**Table 2:** Functional chemical component changes of DGTNs before and after water cooking.

Addition Amount	DGTN 1		DGTN 2		DGTN 3	
	0.5%	2%	0.5%	2%	0.5%	2%
**Chlorophyll *a***	-	-	-	-	-	-
Uncooked (μg/g)	17.37 ± 0.04 ^b^	60.73 ± 1.60 ^e^	13.76 ± 0.37 ^a^	52.36 ± 0.23 ^d^	13.62 ± 0.05 ^a^	45.84 ± 0.06 ^c^
Cooked (μg/g)	14.50 ± 0.10 ^a^	46.74 ± 1.09 ^c^	13.67 ± 0.11 ^a^	44.90 ± 2.40 ^c^	13.06 ± 0.06 ^a^	40.64 ± 0.66 ^b^
Retention rate (%)	83.48 ± 0.73 ^ab^	76.96 ± 0.23 ^a^	99.47 ± 3.52 ^c^	85.77 ± 4.94 ^b^	95.87 ± 0.84 ^c^	88.65 ± 1.32 ^b^
**Chlorophyll *b***			-	-	-	-
Uncooked (μg/g)	13.85 ± 0.05 ^b^	32.46 ± 0.73 ^e^	11.83 ± 0.09 ^a^	28.74 ± 0.29 ^d^	11.40 ± 0.15 ^a^	24.99 ± 0.21 ^c^
Cooked (μg/g)	12.32 ± 0.15 ^b^	24.83 ± 0.40 ^e^	11.15 ± 0.03 ^a^	22.87 ± 0.85 ^d^	10.72 ± 0.00 ^a^	20.64 ± 0.31 ^c^
Retention rate (%)	88.95 ± 0.71 ^c^	76.50 ± 0.49 ^a^	94.25 ± 0.44 ^d^	79.62 ± 3.78 ^ab^	94.10 ± 1.28 ^d^	82.59 ± 1.94 ^b^
**Total chlorophyll**			-	-	-	-
Uncooked (μg/g)	31.22 ± 0.09 ^b^	93.20 ± 2.33 ^e^	25.58 ± 0.46 ^a^	81.10 ± 0.52 ^d^	25.02 ± 0.21 ^a^	70.83 ± 0.15 ^c^
Cooked (μg/g)	26.82 ± 0.05 ^a^	71.56 ± 1.49 ^c^	24.82 ± 0.08 ^a^	67.76 ± 3.25 ^c^	23.78 ± 0.06 ^a^	61.28 ± 0.97 ^b^
Retention rate (%)	85.91 ± 0.01 ^b^	76.80 ± 0.32 ^a^	97.04 ± 2.07 ^c^	83.59 ± 4.54 ^b^	95.07 ± 1.04 ^c^	86.51 ± 1.55 ^b^
**Free phenolic content**	-	-	-	-	-	-
Uncooked (mg/g)	3.18 ± 0.02 ^b^	5.42 ± 0.00 ^d^	2.46 ± 0.03 ^a^	4.92 ± 0.18 ^c^	2.76 ± 0.05 ^a^	4.70 ± 0.22 ^c^
Cooked (mg/g)	1.71 ± 0.06 ^a^	3.41 ± 0.09 ^c^	1.57 ± 0.01 ^a^	3.34 ± 0.01 ^bc^	1.64 ± 0.01 ^a^	3.19 ± 0.10 ^b^
Retention rate (%)	53.76 ± 2.31 ^a^	62.86 ± 1.54 ^b^	63.96 ± 0.43 ^bc^	67.92 ± 2.74 ^c^	59.37 ± 0.69 ^b^	67.82 ± 1.08 ^c^

Different lowercase letters show significant differences (α < 0.05) among the same row.

**Table 3:** Changes in antioxidant activities of GTPs and DGTNs before and after water cooking.

Sample	DPPH/IC_50_ (mg/mL)		ABTS/mmol/g		FRAP/mmol/g	
GTP 1	0.71 ± 0.02 ^a^		2.24 ± 0.17 ^b^		2.28 ± 0.06 ^c^	
GTP 2	0.84 ± 0.00 ^b^		1.50 ± 0.08 ^a^		1.55 ± 0.10 ^b^	
GTP 3	0.87 ± 0.00 ^c^		1.51 ± 0.10 ^a^		1.22 ± 0.01 ^a^	
-	DPPH/IC_50_ (mg/mL)		ABTS/mmol/kg		FRAP/mmol/kg	
-	Uncooked	Cooked	Uncooked	Cooked	Uncooked	Cooked
0.5% DGTN 1	188.5 ± 7.7 ^a^	295.8 ± 3.3 ^a^	7.76 ± 0.00 ^c^	5.22 ± 0.29 ^a^	4.05 ± 0.28 ^a^	3.16 ± 0.02 ^a^
0.5% DGTN 2	199.7 ± 7.6 ^a^	315.6 ± 44.1 ^a^	6.82 ± 0.05 ^a^	6.07 ± 0.16 ^a^	3.69 ± 0.18 ^a^	2.91 ± 0.54 ^a^
0.5% DGTN 3	224.3 ± 4.1 ^b^	288.5 ± 17.1 ^a^	7.14 ± 0.13 ^b^	5.65 ± 0.49 ^a^	3.77 ± 0.11 ^a^	3.43 ± 0.07 ^a^
-	-	-	-	-	-	-
2% DGTN 1	55.7 ± 1.1 ^a^	79.7 ± 6.9 ^a^	24.48 ± 1.03 ^b^	16.64 ± 1.69 ^a^	18.63 ± 1.29 ^a^	12.44 ± 0.06 ^a^
2% DGTN 2	67.7 ± 1.5 ^b^	91.5 ± 1.0 ^b^	22.32 ± 0.68 ^a^	16.06 ± 0.15 ^a^	17.28 ± 0.88 ^a^	11.87 ± 0.85 ^a^
2% DGTN 3	66.0 ± 0.6 ^b^	89.1 ± 2.0 ^ab^	21.05 ± 0.31 ^a^	16.06 ± 0.12 ^a^	17.88 ± 0.28 ^a^	11.85 ± 1.48 ^a^

Different lowercase letters show significant differences (α < 0.05) among the three GTPs and their DGTNs.
